# Right stellate ganglion stimulation modulates arrhythmogenesis in acute left lateral ventricular ischaemia

**DOI:** 10.1093/cvr/cvaf121

**Published:** 2025-07-14

**Authors:** Joseph Hadaya, Bastiaan J D Boukens, Michiel J Janse, Steven Cha, Al-Hassan Dajani, Ronald Challita, Ruben Coronel, Jeffrey L Ardell, Kalyanam Shivkumar, Veronique M F Meijborg

**Affiliations:** UCLA Cardiac Arrhythmia Center and Neurocardiology Research Program of Excellence, David Geffen School of Medicine at UCLA, Los Angeles, CA, USA; Laboratory of Experimental Cardiology, Department of Cardiology, LUMC, Leiden, The Netherlands; Department of Physiology, University of Maastricht, Universiteitssingel 50, P.O. Box 616, Maastricht 6200 MD, The Netherlands; Department of Experimental Cardiology, Amsterdam UMC, University of Amsterdam, Meibergdreef 9, P.O. Box 22660, Amsterdam 1100 DD, The Netherlands; UCLA Cardiac Arrhythmia Center and Neurocardiology Research Program of Excellence, David Geffen School of Medicine at UCLA, Los Angeles, CA, USA; UCLA Cardiac Arrhythmia Center and Neurocardiology Research Program of Excellence, David Geffen School of Medicine at UCLA, Los Angeles, CA, USA; UCLA Cardiac Arrhythmia Center and Neurocardiology Research Program of Excellence, David Geffen School of Medicine at UCLA, Los Angeles, CA, USA; Department of Experimental Cardiology, Amsterdam UMC, University of Amsterdam, Meibergdreef 9, P.O. Box 22660, Amsterdam 1100 DD, The Netherlands; UCLA Cardiac Arrhythmia Center and Neurocardiology Research Program of Excellence, David Geffen School of Medicine at UCLA, Los Angeles, CA, USA; UCLA Cardiac Arrhythmia Center and Neurocardiology Research Program of Excellence, David Geffen School of Medicine at UCLA, Los Angeles, CA, USA; Department of Experimental Cardiology, Amsterdam UMC, University of Amsterdam, Meibergdreef 9, P.O. Box 22660, Amsterdam 1100 DD, The Netherlands; Laboratory for Experimental Cardiology, Department of Cardiology, University Medical Center Utrecht, Heidelberglaan 100, P.O. Box 85500, Utrecht 3508 GA, The Netherlands; Department of Medical Physiology, University Medical Center Utrecht, Utrecht 3584 CX, The Netherlands

**Keywords:** Acute ischaemia, Injury current, Ventricular arrhythmia, Autonomic modulation, Repolarization

## Abstract

**Aims:**

Acute myocardial ischaemia causes fatal arrhythmias as result of a flow of ‘injury current’. Left stellate ganglion stimulation (LSGS) modulates the injury current and is arrhythmogenic during left anterior ventricular wall ischaemia. The role of right stellate ganglion stimulation (RSGS) in arrhythmogenesis is unclear. We hypothesized that RSGS is proarrhythmic during left lateral ventricular wall ischaemia.

**Methods and results:**

In 11 anaesthetized female pigs, ventricular repolarization was measured in unipolar epicardial electrograms from the left lateral ventricular wall. Seven subsequent episodes of acute ischaemia (5 min) were produced by occlusion of the circumflex coronary artery (CX), separated by 20 min of reperfusion. The second occlusion served as a control. After 3 min of ischaemia during the third occlusion, LSGS was initiated for 30 s. In the 4th occlusion, RSGS was performed. After decentralization of both left and right stellate ganglia and vagal nerves, LSGS and RSGS were initiated (6th and 7th occlusions). RSGS during ischaemia was more arrhythmogenic than LSGS or control with more spontaneous ventricular premature beats (3–5 min of ischaemia) and two instances of ventricular fibrillation. The LSGS-induced effect on repolarization was absent in myocardium that had been ischaemic for 3 min by CX occlusion.

**Conclusions:**

LSGS-induced repolarization shortening is absent in ischaemic myocardium. RSGS was more arrhythmogenic following CX occlusion than LSGS or control. These data demonstrate that the arrhythmogenic influence of RSGS or LSGS is contingent on the location of ischaemic zone supporting the clinical findings that bilateral sympathectomy is superior to left sympathectomy alone.

## Graphical Abstract

**Graphical Abstract:**
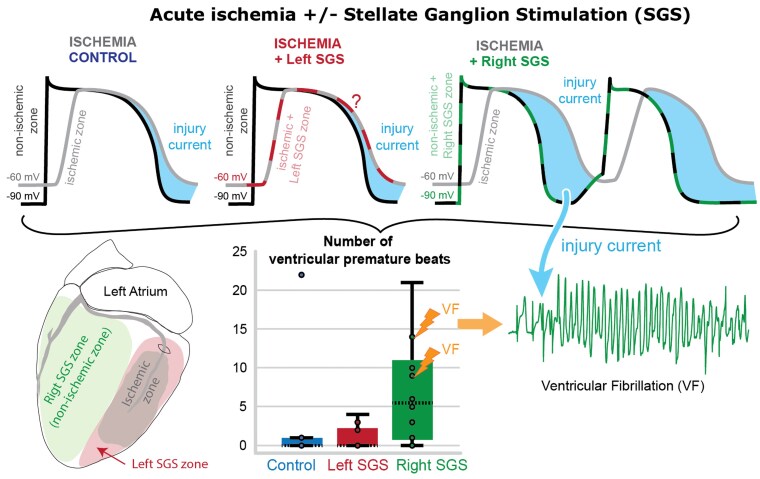

**Time of primary review: 49 days**

## 1. Introduction

Acute myocardial ischaemia is one of the leading causes of death in the developed world.^1^ During acute ischaemia, an intracellular ‘injury current’ flows towards the surrounding non-ischaemic myocardium. This ‘injury’ current can become large enough to re-excite the non-ischaemic myocardium adjacent to the ischaemic border and causes a spontaneous premature beat,^2,3^ which may initiate ventricular tachycardia or fibrillation leading to death.

**Figure 1:**
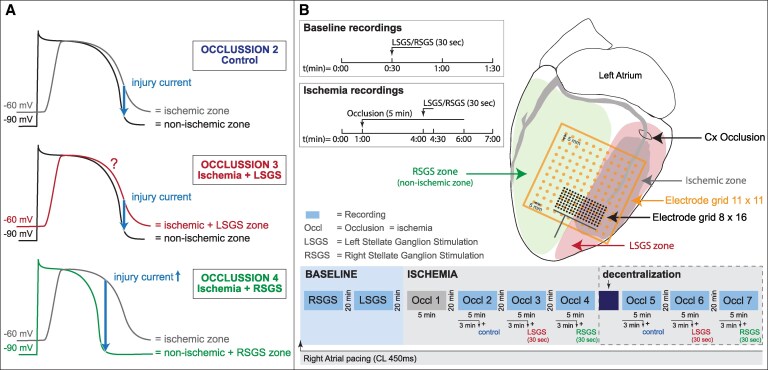
(*A*) Hypothesized mechanism of injury current in the different conditions; control occlusion (upper panel), occlusion with LSGS (middle panel), and occlusion with RSGS (lower panel). In each condition, action potentials are shown from the ischaemic and non-ischaemic zones expected to occur 3:30–5:00 min of ischaemia. Note the larger injury current in the setting of occlusion with RSGS, which is expected to result in a higher incidence of ventricular premature beats. (*B*) Schematic overview of the measurement protocol. The heart in anterolateral view shows the ischaemic zone and the zones effected by right and left stellate ganglion stimulation (RSGS and LSGS zones, respectively). The electrode grid is positioned overlying both ischaemic/LSGS and non-ischaemic/RSGS zones. Lower panel: demonstrates the recording protocol; baseline recordings of RSGS and LSGS, repetitive occlusions before and after decentralization. The left upper boxes indicate specific timing-schemes of recordings. LSGS and RSGS, left and right stellate ganglion stimulation; Cx, circumflex artery; Occl, occlusion.

Shortening of repolarization in the non-ischaemic tissue may increase the ‘injury current’. Left stellate ganglion stimulation (LSGS) causes a shortening of repolarization by about 30 ms in the left lateral wall,^4^ and increases the incidence of ventricular premature beats (VPBs) during ischaemia of the anterior left ventricular wall compared to ischaemia without stellate ganglion stimulation.^5^ We hypothesized that the reverse is also true and that the effects of right stellate ganglion stimulation (RSGS) during ischaemia of the lateral wall caused by occlusion of the circumflex coronary artery exerts similar proarrhythmic effects. RSGS causes regional shortening of repolarization in the anterior (non-ischaemic) wall and increases the injury current flowing from the lateral (ischaemic) tissue towards the anterior (non-ischaemic) tissue. Thus, more VPBs are to be expected than during ischaemia with LSGS or without stellate ganglion stimulation. *Figure 1A* illustrates the proposed mechanism of injury current underlying the hypothesis. In previous studies, we found that dispersion in repolarization was increased after decentralization,^1^ and that during anterior ventricular wall ischaemia, the incidence of ventricular tachycardia or fibrillation was higher after decentralization.^2^ Therefore, we explored the effect of stellate stimulation during left lateral wall ischaemia before and after decentralization. The results demonstrate that RSGS is proarrhythmic during acute lateral wall ischaemia and imply that right sympathectomy should be considered as an antiarrhythmic option.

## 2. Methods

Animal handling and care followed the recommendations of the National Institutes of Health Guide for the Care and Use of Laboratory Animals and the University of California, Los Angeles, Institutional Animal Care and Use Committees. Animal protocols were approved by the Chancellor’s Animal Research Committee, University of California, Los Angeles.

### 2.1 Animal preparation

Female Yorkshire pigs (*n* = 11, weighing 45–64 kg) were premedicated with intramuscular tiletamine–zolazepam (4–6 mg/kg), intubated, and mechanically ventilated. General anaesthesia during surgical procedures was maintained with isoflurane (2–3%, inhalation) and with intermittent boluses of fentanyl citrate (20–30 µg/kg, iv) to maintain analgesia. Animals underwent median sternotomy to expose the heart and the sympathetic chain in the posterior thorax. Following completions of all preparatory surgical procedures, anaesthesia was switched to solely alpha-chloralose (20–30 mg/kg/h) for the remainder of the experimental protocols. Intravenous access through the femoral veins allowed administration of drugs. Monitoring during the experimental protocols included surface electrocardiograms, arterial blood pressure, temperature, oxygen saturation, and end-tidal carbon dioxide.

The left and right stellate ganglia were electrically stimulated for 30 s by using a bipolar platinum electrode connected to a Grass stimulator (S88, Grass Technologies, West Warwick, RI, USA) connected to a SIU6 constant current stimulus (4 Hz) isolation unit, as published previously.^6^ Aortic pressure was monitored by using a 5 Fr pigtail pressure catheter connected to a MPVS Ultra processor (Millar Instruments, Inc., Houston, TX, USA) inserted via the carotid artery. For LSGS, an increase in aortic pressure of 30% was targeted; for RSGS, a heart rate increase of 30% with a minimum cycle length of 500 ms was targeted.

The first (or first two) obtuse marginals of the circumflex coronary artery were prepared free, and a silk ligature was passed underneath the branch(es). The threads of the ligature were passed through a rubber tourniquet, and temporary coronary occlusion was performed by pulling the ligatures and clamping the tourniquet. Occlusions lasted 5 min and were separated by a 20 min reperfusion interval. The electrophysiological changes during a first occlusion are known to be different from those during a second occlusion, whereas the changes in the second occlusion and subsequent occlusions are reproducible.^7–9^ We therefore used the second occlusion as a control occlusion and the following occlusions to compare sympathetic interventions. Animals were euthanized by a lethal dose of potassium chloride and sodium pentobarbital (100 mg/kg).

### 2.2 Electrophysiological recordings

Recordings were made from 121 epicardial sites via an 11 × 11 electrode grid (5 × 5 cm, interelectrode distance 5 mm, as used before^5^) or from 128 epicardial sites 8 × 16 electrode grid (14 × 30 mm, interelectrode distance 2 mm) allowing high density mapping across the ischaemic border. The electrode grids covered both the ischaemic and normal myocardium, covering the anticipated border between areas influenced by the left and right stellate stimulation, respectively (see *Figure 1B*). The reference electrode was positioned subcutaneously at the thoracic incision site. A bipolar stimulus electrode was attached to the right atrium. The right atrium was stimulated at a cycle length of 400–450 ms and was kept constant between different measurement conditions. Unipolar electrograms from the high density electrode grid were recorded via a multichannel data acquisition system {8 × 16 grid: AlphaLab SnR—Alpha Omega, Nazareth, Israel, 16 bit, 38.147 μV bit resolution, sampling frequency of 1375 Hz [bandwidth DC—400 Hz]; 11 × 11 grid: BioSemi, Amsterdam, 24 bit dynamic range, 122.07 nV LSB, total noise 0.5 mV, sampling frequency of 2048 Hz [bandwidth (−3 dB) DC—400 Hz]}. Body surface electrocardiograms were recorded for monitoring the heart condition during the experiment. In each experiment, recordings were made throughout four successive ischaemic episodes: the control (2nd) occlusion, the 3rd occlusion during which LSGS for 30 s was initiated after 3 min of ischaemia, and the 4th occlusion during which RSGS for 30 s was initiated after 3 min of ischaemia. In a subset of experiments (*n* = 4), similar ischaemic episodes were repeated following decentralization of both left and right stellate ganglia by cutting the caudal extension of the ganglia: control (5th) occlusion, occlusion with 30 s LSGS (6th), and occlusion with 30 s RSGS (7th) (see *Figure 1B*). Electrical signal analysis was performed offline using custom-made data analysis software based on MATLAB (MathWorks Inc., Natick, MA, USA) as published previously.^10^ Repolarization times were measured as the interval between the reference time (onset of the q wave using all electrocardiograms) and the time of the maximum positive slope of the T wave in the local electrograms.^11^

### 2.3 Occurrence of VPBs and arrhythmias

The occurrence of VPBs was counted during each occlusion at 3–5 min of ischaemia or until a ventricular arrhythmia was induced. A single VPB was counted as 1, a doublet as 2, and a triplet as 3. In case of ventricular tachycardia or fibrillation (VT/VF), defibrillation was applied and, after 20 min of recovery, we continued with the following occlusion.

### 2.4 Statistics

Continuous variables were presented as mean ± SD if normally distributed and in median (25th–75th percentile) if not normally distributed. The difference in number of VPBs was tested using a Friedman test, followed by *post hoc* testing. A *P*-value of ≤0.05 was considered statistically significant.

## 3. Results

### 3.1 Electrophysiological effect of LSGS within the ischaemic zone

**Figure 2:**
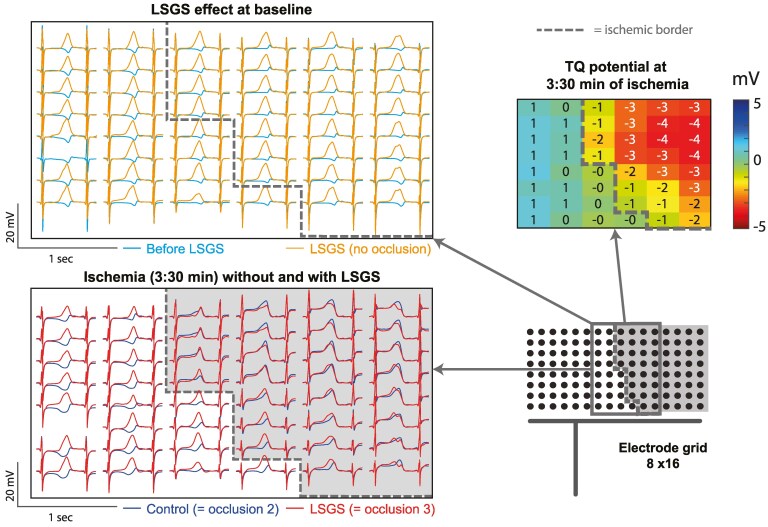
Example of a comparison of unipolar electrograms showing the LSGS effect under baseline condition (upper panel: before LSGS vs. 30 s of LSGS) and at 3:30 min of ischaemia without and with left stellate ganglion stimulation [lower panel: control (occlusion 2) vs. LSGS (occlusion 3)]. A section of the electrode grid is shown, outlined in the schematic configuration (right lower panel). The section overlaps the border between ischaemic and non-ischaemic zones, indicated by the interrupted line. Upper right panel: TQ-potential map of the same electrode section measured at 3:30 min of ischaemia during control occlusion.

*Figure 2* shows the LSGS-induced effect on the unipolar electrograms before and during ischaemia in a section of the electrode grid outlined in the schematic configuration (lower right panel). As expected,^4^ before ischaemia (baseline condition), LSGS causes a shortening of repolarization of about 60 ms, which is visible by a leftward shift of the T-wave upstroke (left upper panel). During acute ischaemia, TQ depression is observed in part of the electrode grid indicating the ischaemic zone (TQ potentials ≤ −1, upper right panel). The comparison of unipolar electrograms (lower left panel) of an occlusion without (control, occlusion 2) and with LSGS (occlusion 3) demonstrates that within the ischaemic zone (electrograms with ST-elevation, right to the dashed line), the signals during the 2nd and 3rd occlusions are virtually identical, indicating that LSGS has no longer an effect on ischaemic myocardium. Outside the ischaemic zone (left to the dashed line), there is still a clear shortening of repolarization present (leftward shifted positive slope of the red signals). This was present in all cases (*n* = 2) in which the grid covers also a non-ischaemic zone with innervation of LSGS. The upper right panel shows a map of TQ potentials, which are negative in the ischaemic zone.

In some case, the electrode grid did not fully cover the ischaemic zone (one experiment), or the area affected by LSGS during the testing period (seven experiments) out of 11 experiments. In the remaining three experiments, repolarization was not shortened within the ischaemic area (selected based on TQ depression) when ischaemia was combined with LSGS compared to ischaemia without LSGS (*Table 1*, OCCL3 vs. OCCL2, respectively), whereas in this same area, repolarization had shortened upon LSGS before ischaemia was induced (baseline). Thus, the LSGS effects on repolarization are no longer present after 3 min of ischaemia.

**Table 1:** Changes in repolarization time (delta RT: RT at 30 s of LSGS/control minus RT before LSGS/control) of a selection of electrodes (*n*) within the anticipated ischaemic area during baseline condition (LSGS before ischaemia), occlusion 2 (OCCL2, ischaemia without LSGS), and occlusion 3 (OCCL3, ischaemia with LSGS)

Delta RT (ms, mean ± SD)			
	Baseline (no ischaemia + LSGS)	OCCL 2 (ischaemia control)	OCCL 3 (ischaemia + LSGS)
Pig #2 (***n*** = 21)	−11 ± 4	−3 ± 3	5 ± 4
Pig #4 (***n*** = 31)	−12 ± 7	13 ± 7	8 ± 10
Pig #6 (***n*** = 28)	−86 ± 9	−5 ± 9	−1 ± 34

Data are shown as mean and standard deviation (SD) per pig in which the electrode grid covered both ischaemic and LSGS-affected tissue.

### 3.2 Arrhythmogenicity during ischaemia while stimulating the stellate

**Figure 3:**
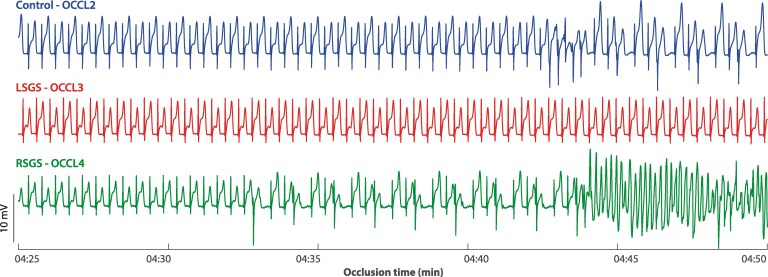
Example of unipolar electrograms from an electrode in the ischaemia region during control occlusion (control—OCCL2) and during occlusion with left (LSGS—OCCL3) or right stellate ganglion stimulation (RSGS—OCCL4), at 4:25 min of ischaemia. During occlusion with RSGS, ventricular fibrillation is induced that could not be converted.

In *Figure 3*, unipolar electrograms from one electrode in the ischaemic region are shown during the control occlusion and during the 3rd and 4th occlusions when respectively LSGS or RSGS was applied. During the occlusion with RSGS, VPBs occurred after which spontaneous ventricular fibrillation was initiated. Although there are VPBs during the control occlusion, the arrhythmogenic effect of RSGS is highlighted by more VPBs and the onset of ventricular fibrillation.

**Figure 4:**
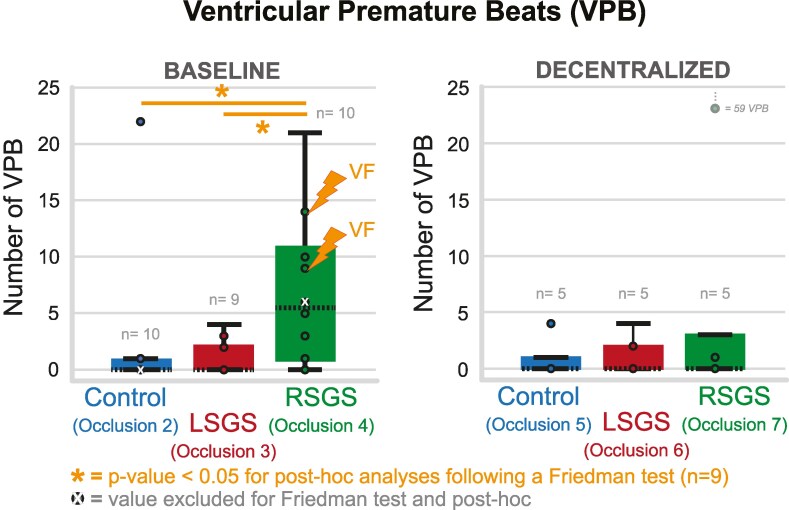
Boxplots of number of ventricular premature beats (VPBs) occurring during the different occlusions (counted 3:00–5:00 min of ischaemia) before (left) and after decentralization of stellates and vagal nerves (right). In two cases, ventricular fibrillation (VF) is initiated during the occlusion with RSGS.

In one experiment, the RSGS effects on heart rate and repolarization (before ischaemia) were absent and the data of these interventions were excluded from analyses of VPB counts. In the other 10 experiments, the number of VPBs was counted between 3 and 5 min of ischaemia, both before and after decentralization of the stellate ganglia and vagal nerves. Before decentralization, the number of VPBs during RSGS was significantly higher (*P* = 0.01, Friedman test, *n* = 9, one experiment lacks an occlusion 3 with LSGS due to absent LSGS effect) compared to control and LSGS (*Figure 4*, left panel). In two cases, ventricular fibrillation occurred during the 4th occlusion (ischaemia with RSGS), which could not be converted and hindered further recordings. After decentralization (*n* = 5), the number of VPBs was not significantly different between the different occlusions (*Figure 4*, right panel).

## 4. Discussion

The two main results of this study are: (i) the LSGS-induced effect on repolarization is absent in myocardium that has been ischaemic for 3 min; and (ii) RSGS applied during left lateral ventricular wall ischaemia (by CX occlusion) is more arrhythmogenic compared to LSGS applied during ischaemia or ischaemia alone.

Our previous observations^5^ demonstrated that LSGS is more arrhythmogenic compared to no LSGS when applied during anterior ventricular wall ischaemia. These observations also supported the concept of the increased ‘current of injury’ induced by the abrupt shortening of the action potential duration (caused by stellate ganglion stimulation) in the healthy myocardium adjacent to the ischaemic myocardium and leading to a focal origin of VPBs in that tissue. We now confirm that the reciprocal condition can be induced by RSGS during ischaemia of the left lateral wall. Therefore, left and right stellate ganglion stimulation are both arrhythmogenic depending on the localization of the ischaemic zone. Following decentralization, the number of VPBs did not differ between the conditions and support that bilateral sympathectomy leads to reduced arrhythmogenicity.

In addition, we documented that LSGS during left lateral ischaemia did not result in repolarization changes inside the ischaemic tissue. This can be the result of one of two mechanisms: (i) the neural tissue is equally depolarized as the myocardial tissue by the increase in extracellular potassium concentration causing neural inexcitability, or (ii) the neurotransmitter stores are depleted during the first minutes of ischaemia. Neural tissue is relatively resistant to ischaemia and neural fibres typically continue to conduct impulses even during depolarization of the resting membrane.^12^ Chan *et al*.^13^ have shown with continuous local needle recordings that extracellular catecholamine levels show an immediate local increase following regional ischaemia. This makes it plausible that left lateral wall ischaemia depletes local neurotransmitter stores of neural fibres in this region (e.g. LSG-fibres, an RSGS effect was not observed in this region) immediately after initiation of ischaemia, and that, later, LSGS no longer leads to its typical changes on repolarization. The same theory may hold for a depletion of neurogenic stores of RSG-fibres following anterior wall ischaemia.

### 4.1 Methodological considerations

In contrast to our findings during occlusion of the left anterior descending artery (leading to anterior wall ischaemia),^5^ we did not observe that VPBs were preceded by negative T waves in the ischaemic myocardium. This could have been due to the small dimensions of the high density 8 × 16 electrode grid (14 × 30 mm), which also accounted for the fact that in some experiments, the grid did not cover the area affected by LSGS (seven experiments) or the ischaemic zone (one experiment). In the experiments with the larger 11 × 11 electrode grid (5 × 5 cm), we also did not observe negative T waves immediately preceding VPBs. The VPBs thus likely originated outside the area covered with the 11 × 11 grid, i.e. from the basal, apical, or posterior walls. These regions are more difficult to approach with the electrode grid. Quantification of the T-wave potential (as reported in Boukens *et al*.^5^) was therefore also irrelevant. However, it has been solidly established that the origin of VPBs in early regional ischaemia depends on the ‘injury current’.^2,3^ Nonetheless, we conclude that the number of VPBs was larger when RSGS was applied during acute ischaemia of the left lateral wall compared to control or LSGS. The order of different conditions (occlusion with no, left or right stellate ganglion stimulation) was always kept the same to allow measurements under all conditions without losing the animal in between due to unconvertable arrhythmias (expected to be most likely in the setting of ischaemia + RSGS).

It has been shown that T-wave alternans (TWA) is associated with arrhythmia incidence.^14^ Qu *et al*.^15^ have tested the hypothesis that TWA (especially of discordant type) creates a substrate for re-entry, because it results in a local dispersion in repolarization, a prerequisite for re-entry. In the current paper, we are focused on the mechanism of the trigger (VPBs) initiating the arrhythmia, rather than the underlying substrate and occurrences of re-entry itself. Nevertheless, we surmised that TWA would more likely occur during occlusion with RSGS compared to occlusion without RSGS. However, the quantification of TWA in our data was hampered, because of continuous and highly dynamic changes in the T wave caused by RSGS and LSGS^4^ and ischaemia itself. The quantification resulted in only some incidental findings (see supplemental data).

### 4.2 Clinical relevance

Together, our previous^5^ and present studies show that LSGS is arrhythmogenic during anterior wall ischaemia caused by occlusion of the left anterior coronary artery, and that RSGS is not, whereas during circumflex occlusion, the RSGS is more arrhythmogenic. There has been considerable controversy over the antiarrhythmic effects of left vs. bilateral sympathetic denervation.^16,17^ Our data indicate that the location of the ischaemic zone determines whether the left or the right stellate ganglion is arrhythmogenic. Our data suggest that bilateral sympathetic cardiac denervation is superior to left denervation alone in patients with structural heart disease.^17–19^

Translational perspective Cardiac sympathetic denervation is a promising therapy for reducing arrhythmias during acute cardiac ischaemia. There has been considerable controversy over the antiarrhythmic effects of left vs. bilateral sympathetic denervation. Our data indicate that the location of the ischaemic zone determines whether the left or the right stellate ganglion is arrhythmogenic and support the clinical findings that bilateral sympathetic cardiac denervation is superior to left denervation alone in patients with structural heart disease.

## Supplementary Material

cvaf121_Supplementary_Data

## Data Availability

The data underlying this article will be shared on reasonable request to the corresponding author.
