# Supplementary material for: Right stellate ganglion stimulation modulates arrhythmogenesis in acute left lateral ventricular ischaemia
Source: Cardiovasc Res. 2025 Jul 14;121(9):1385–91. doi: 10.1093/cvr/cvaf121 (PMC12352303; doi:10.1093/cvr/cvaf121)
Supplement: cvaf121_Supplementary_Data [file cvaf121_supplementary_data.docx]

# **Supplemental data**

For “Right stellate ganglion stimulation modulates arrhythmogenesis in acute left lateral ventricular ischemia”

Short title: sympathetic stimulation during acute ischemia

Joseph E. Hadaya ([JHadaya@mednet.ucla.edu](mailto:JHadaya@mednet.ucla.edu)) ^1^;

Bastiaan J.D.Boukens ([b.boukens@maastrichtuniversity.nl](mailto:b.boukens@maastrichtuniversity.nl)) ^2,3^;

Michiel J.Janse ([m.j.janse@amsterdamumc.nl](mailto:m.j.janse@amsterdamumc.nl)) ^4^;

Steven Cha ([sjcha023@gmail.com](mailto:sjcha023@gmail.com)) ^1^;

Al-Hassan Dajani ([adajani@mednet.ucla.edu](mailto:adajani@mednet.ucla.edu)) ^1^;

Ronald Challita (Ron.challita@gmail.com) ^1^;

Ruben Coronel ([rubencoronel@gmail.com](mailto:rubencoronel@gmail.com)) ^4^;

Jeffrey L. Ardell ([JArdell@mednet.ucla.edu](mailto:JArdell@mednet.ucla.edu)) ^1^;

Kalyanam Shivkumar ([KShivkumar@mednet.ucla.edu](mailto:KShivkumar@mednet.ucla.edu)) ^1^;

Veronique M.F. Meijborg ([veromeijborg@gmail.com](mailto:veromeijborg@gmail.com)) ^4,5,6^.

*^1^ UCLA Cardiac Arrhythmia Center, Los Angeles, CA, USA.(JH, SC, AD, NC, JLA, KS).*

*^2^ Laboratory of Experimental Cardiology, Department of Cardiology, LUMC, Leiden, The Netherlands (BJDB)*

*^3^ Department of Physiology, University of Maastricht, Universiteitssingel 50, PO Box 616, 6200 MD Maastricht, The Netherlands*

*^4^ Department of Experimental Cardiology, Amsterdam UMC, University of Amsterdam, Meibergdreef 9, P.O.Box 22660, 1100 DD Amsterdam , The Netherlands (MJJ, RC, VMFM);*

^5^ Laboratory for Experimental Cardiology, Department of Cardiology, University Medical Center Utrecht, Utrecht, the Netherlands (VMFM)

^6^ Department of Medical Physiology University Medical Center Utrecht, 3584 CX Utrecht, The Netherlands (VMFM)

**Corresponding author:**

Veronique M.F. Meijborg, Laboratory for Experimental Cardiology, Department of Cardiology, University Medical Center Utrecht, Heidelberglaan 100, P.O. Box 85500, 3508 GA, Utrecht, the Netherlands. Tel. no.: +31 88 75 57654

**Supplemental Figure 1**: Electrograms showing TWA: OCCL2-CTRL vs OCCL4-RSGS at 3:28 min of ischemia.

Quantification of T-wave alternans

T-wave alternans (TWA) is an intriguing concept that has been shown to be associated with arrhythmia incidence^1,2^. We surmised that TWA would more likely occur during occlusion with RSGS compared to occlusion without RSGS. The quantification of TWA in our data was hampered, because of continuous and highly dynamic changes in the T-wave caused by RSGS and LSGS^3^. These dynamic changes interfere with the alternating and dynamic T-wave changes (TWA) caused by ongoing ischemia itself. Also, the occurrence of spontaneous VPBs (which occurred more often in OCCL4) hampers the quantification of TWA, because the VPB-induced changes in cycle length influences the repolarization and thus the level of TWA. The VPBs did not occur strictly at the same moment within the 3:00 – 5:00 min of ischemia between the different occlusions (with or without SGS) as well as between animals. It was therefore virtually impossible to ‘standardize’ TWA analysis in order to properly compare different occlusions.

Nonetheless, we observed more TWA in OCCL4-RSGS vs OCCL2-CTRL (n=3) but at different moments during ischemia. A marked example is shown in Supplemental Figure 1 demonstrating more TWA in OCCL4-RSGS vs OCCL2-CTRL at 3:28 min during ischemia (i.e. 28 sec of RSGS in OCCL4). In this particular case, we counted 6 VPBs during OCCL4 without occurrence of VF, compared to zero VPBs during OCCL2. These obvious TWA differences were incidental findings and therefore we cannot draw definitive conclusions from them.

References

1. Nearing BD, Oesterle SN, Verrier RL. Quantification of ischaemia induced vulnerability by precordial T wave alternans analysis in dog and human. *Cardiovasc Res* 1994;**28**:1440–1449.

2. Qu Z, Garfinkel A, Chen P-S, Weiss JN. Mechanisms of Discordant Alternans and Induction of Reentry in Simulated Cardiac Tissue. *Circulation* 2000;**102**:1664–1670.

3. Meijborg VMF, Boukens BJD, Janse MJ, Salavatian S, Dacey MJ, Yoshie K, Opthof T, Swid MA, Hoang JD, Hanna P, Ardell J, Shivkumar K, Coronel R. Stellate ganglion stimulation causes spatiotemporal changes in ventricular repolarization in pig. *Heart Rhythm* 2020;**17**:795–803.
